# Risk areas for tuberculosis among children and their inequalities in a city from Southeast Brazil

**DOI:** 10.1186/s12887-020-02364-7

**Published:** 2020-10-06

**Authors:** Yan Mathias Alves, Thaís Zamboni Berra, Luana Seles Alves, Ivaneliza Simionato de Assis, Marcos Augusto Moraes Arcoverde, Antonio Carlos Vieira Ramos, Luiz Henrique Arroyo, Laura Terenciani Campoy, Alexandre Tadashi Inomata Bruce, Felipe Lima dos Santos, Ludmilla Leidianne Limirio Souza, Juliane de Almeida Crispim, Ricardo Alexandre Arcêncio

**Affiliations:** 1 Public Health Nursing Graduate Program, University of São Paulo at Ribeirão Preto College of Nursing, Avenida dos Bandeirantes, 3900, Monte Alegre, Ribeirão Preto, São Paulo, 14040-902 Brazil; 2 União Dinâmica Cataratas College, Foz do Iguazu, Paraná, Brazil; 3 Western Paraná State University, Foz do Iguaçu, Paraná, Brazil; 4 Inter-institutional Doctoral Program in Nursing, University of São Paulo at Ribeirão Preto College of Nursing, Ribeirão Preto, São Paulo, Brazil; 5 University of São Paulo at Ribeirão Preto College of Nursing, Ribeirão Preto, São Paulo, Brazil

**Keywords:** Tuberculosis, Spatial analysis, Social vulnerability

## Abstract

**Background:**

The objective of the study was to identify areas of risk for the appearance of tuberculosis in children and their association with social inequalities in a municipality in southeastern Brazil.

**Methods:**

Ecological study conducted in Ribeirão Preto, Brazil. To identify areas of spatial risk for tuberculosis in children, we used spatial scanning statistics. To analyze the association of cases of childhood tuberculosis with social vulnerability, we used the Social Vulnerability Index of São Paulo, and four explanatory statistical models were listed.

**Results:**

There were 96 cases of childhood tuberculosis, of which 90 were geocoded through a process of converting addresses to geographic coordinates. A risk area was identified in the municipality, where children under 15 years old have 3.14 times greater risk of contracting tuberculosis than those living outside this area. The variables identified as risk factors were: number of private and collective households, proportion of children aged 0 to 5 years in the population, proportion of households without per capita income, and the proportion of private households with monthly nominal incomes of up to one quarter of wage minimums. The variables identified as protection factors were the proportion of women under the age of 30 years responsible for the household under and women responsible for the household with an average income over BRL 2344.

**Conclusion:**

The study showed areas of risk for the occurrence of tuberculosis in children. The study is in line with the End TB Strategy and the 2030 Agenda, which aim to support strategic actions and, therefore, save the lives of children through the systematic, intensified, and comprehensive identification of children with tuberculosis respiratory symptoms in the community.

## Background

Tuberculosis (TB) is a serious global health problem that remains a challenge for public health policies [1]. According to the World Health Organization (WHO) in 2018, there were approximately 10 million new cases of TB in the world; of those, 57% were men, 32% were women, and 11% were children under the age of 15 years. During 2018 in Brazil, more than 73,000 new TB cases were registered, including almost 14,000 cases of TB retreatment and more than 500 cases of multidrug resistant TB [2].

In addition to these alarming data, TB is responsible for 130,000 deaths per year in children, and it is estimated that there are approximately 1 million cases of TB in the world’s child population (about 11,000 cases in Brazil), which means that TB ranks as one of the top 10 causes of death in children worldwide [2].

The main challenge related to childhood TB is the diagnosis, which is made difficult by the absence of a test that can be considered the gold standard. However, the confirmation of the disease in the pediatric age group is often a sentinel event that signals the presence of an adult with TB in the child’s household [3].

The diagnostic techniques classically used in adults have low sensitivity and specificity when used for children, and confirmation by bacteriological identification is not always possible. In addition, many times treatment has been carried out without isolation of mycobacteria, based on a triad referring to the clinical and radiological picture, positivity of the tuberculin test, and contact with an adult with bacilliferous TB [3].

The low positivity of bacteriological tests can be explained by the fact that, in general, TB in children is paucibacillary and, therefore, most of the time they are unable to voluntarily expectorate sputum. An alternative method for obtaining sputum is gastric lavage collection, a relatively invasive method that requires a 3-day hospitalization [3].

Considering the singularities of the TB diagnosis in children, the Brazilian Ministry of Health recommends that the diagnosis of pulmonary TB in children and adolescents (negative for bacilloscopy or undetected in rapid molecular test for TB) be carried out based on scoring or a score system that values clinical, radiological, and epidemiological data and does not require bacteriological confirmation. The use of the score provides early diagnosis and therapeutic intervention even in basic health units, without the need for more sophisticated complementary tests and/or professionals specializing in the disease [4].

According to the WHO, there are economic, social, cultural, ethnic/racial, psychological, and behavioral factors that interfere with the onset of diseases, and these factors are attributed to the persistence in the population of infectious diseases, such as TB, even in the present day. Studies reinforce the assumption that social vulnerability may be associated with the occurrence and distribution of TB in the community [5, 6].

In general, most studies have analyzed the behavior of TB in the general population and little emphasis has been placed on children; identifying areas where children fall ill with TB and their social determinants is an important knowledge gap. This study aimed to identify areas of risk for the occurrence of TB in children in Ribeirão Preto, a city in Southeast Brazil, and the relationship to the social inequalities for that area.

## Methods

**Fig. 1:**
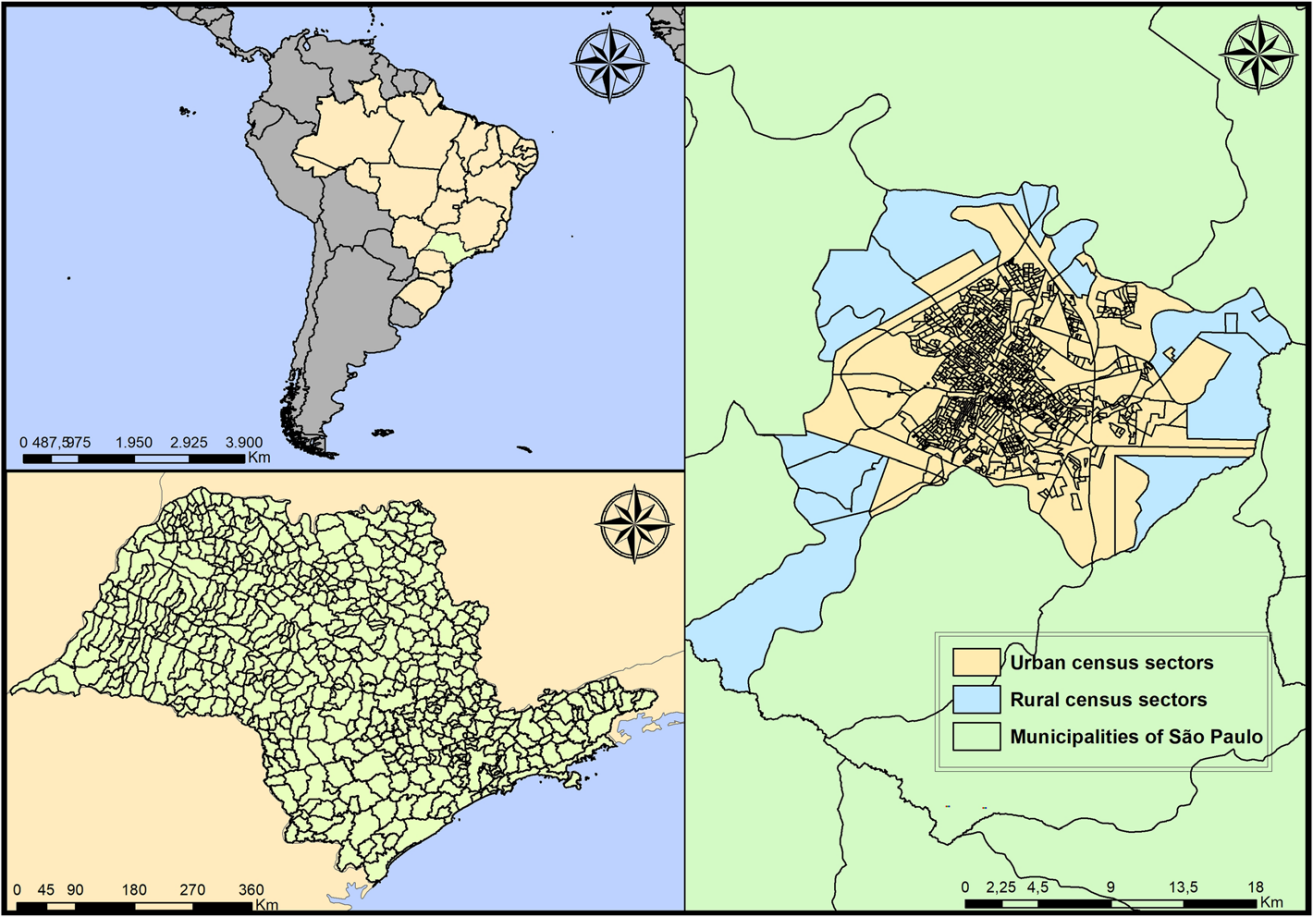
Geographic location of Ribeirão Preto, São Paulo, Brazil

This ecological study was carried out in Ribeirão Preto (Fig. 1), a municipality in the interior of the state of São Paulo, Brazil, located at 47°48′24“W longitude and 21°10’42”S latitude, 314 km from the capital of the state of São Paulo. It has an area of approximately 650 km^2^ and a high population density, with 995.3 inhabitants/km^2^. It also has an estimated population of 647,862 inhabitants in 2010, of which 99.7% live in urban areas [7].

The study population comprised children diagnosed with TB and reported on TBWeb, a TB case reporting system used in the state of São Paulo, from 2006 through 2017. All confirmed cases were considered in children under the age of 15 years (age division carried out by the WHO in which cases of childhood TB are considered to have occurred in children under 15 years old; this age range is considered a priority group for TB treatment) living in Ribeirão Preto with only one registration per person. It is worth noting that cases of latent TB in children under 15 years of age were not included in the study, only those that at the time of notification were active.

First, we performed exploratory analysis to characterize the profile of the cases. This step was carried out using discrete statistics where absolute and relative frequencies of the variables found in the TBWeb notification form were calculated using the IBM SPSS Statistics version 25.0 software.

To verify how childhood TB cases have behaved over the years, we grouped the notified cases were grouped by month of notification and the monthly incidence rate was calculated. To estimate the time trend over the study period measuring cases of TB in children and calculated monthly rates, we used the decomposition method called Seasonal-Trend by Loess (STL), which is based on a locally weighted regression [8]. Graphs for these cases, monthly incidence rates, and time trend estimations were all performed using R studio software.

It was first necessary to perform the geocoding of the notified cases of children with TB. This process consisted of converting the residential addresses of the cases (obtained through the TBWeb notification form) into geographic coordinates (latitude and longitude) through the Google Earth Pro tool for spatial analysis.

To identify areas of spatial risk for TB in children, we used the technique of spatial scanning statistics using SatScan® version 9.5 software; we only included the cases in which it was possible to perform the geocoding of their addresses. As a criterion, we used a discrete Poisson model, without geographical overlapping of clusters and with a circular shape, 999 repetitions, and size of the exposed population stipulated by the Gini coefficient [9]. For statistically significant clusters, *p* < 0.05 was adopted and the 95% confidence interval (CI) was estimated.

To analyze the association of cases of childhood TB with social vulnerability, we use the Social Vulnerability Index of São Paulo (SVI-SP), which classifies the census sections of the municipalities of São Paulo into social vulnerability groups (SVG). These indexes were constructed from variables of the 2010 Brazilian Census of demographic and socioeconomic dimensions, which are in public domain and can be accessed and downloaded through the website (https://ipvs.seade.gov.br/view/index.php?selLoc=0&selTpLoc=2&prodCod=2).

In the analyses, the variables were tested for variance inflation factors (VIFs). A value greater than 10 was adopted as indicative of multicollinearity [10]. After this selection, the remaining variables were dichotomized by their own medians, assigning 1 for values less than or equal to the median and 2 for values greater than the median.

Considering the dependent variable (total number of children with TB), four explanatory statistical models were listed with the following probability distributions: Poisson, negative binomial (BN), zero-inflated Poisson (ZIP), and zero-inflated negative binomial (ZINB). The objective of using four different models was to verify the most adequate model in view of the nature of the data used [11].

The best model was weighted from the lowest values of the Akaike information criterion (AIC) [12]. For the model with the best comparison parameters, the odds ratio (OR) and the respective 95% CIs were calculated. A 5% type I error was fixed as statistically significant (*p* < 0.05).

## Results

There were 98 cases of TB reported in children in the municipality between 2006 and 2017; the children were between 2 months and 14 years old, with a mean age of 8 years and median of 9 years. It is important to note that of these 98 cases, in 65 cases (66.3%) at least one index case (adult) was reported living in the same home as the child.

Table 1 shows the epidemiological profile of cases of childhood TB reported in Ribeirão Preto during the study period. The highest percentage was noted for male children (*n* = 55, 56.1%) and those between 11 and 14 years of age (*n* = 43, 43.9%), but it was noteworthy for all cases of children between 0 and 5 years of age (*n* = 29, 29.6%). The majority of cases occurred in children of white race/color (*n* = 39, 39.8%), with 4–7 years of study (*n* = 16, 16.3%).

**Table 1:** Clinical and epidemiological profile of tuberculosis cases in children, Ribeirão Preto, São Paulo, Brazil (2006–2017)

Variables	N (98)	%
**Age (years)**		
**0–5**	**29**	**29,6**
**6–10**	**26**	**26.5**
**11–14**	**43**	**43.9**
**Gender**		
**Male**	**55**	**56.1**
**Female**	**43**	**43.9**
**Race**		
**White**	**39**	**39.8**
**Brown**	**27**	**27.6**
**Black**	**8**	**8.2**
**Ignored**	**24**	**24.5**
**Years of study**		
**1–3**	**10**	**10.2**
**4–7**	**16**	**16.3**
**8–11**	**13**	**13.3**
**12–14**	**1**	**1.0**
**Ignored**	**37**	**37.7**
**Case type**		
**New**	**89**	**90.8**
**Relapse**	**9**	**9.2**
**Classification**		
**Pulmonary**	**74**	**75.5**
**Extrapulmonary**	**14**	**14.3**
**Concomitant**	**7**	**7.1**
**Disseminated**	**3**	**3.1**
**Discovery method**		
**Outpatient demand**	**48**	**49,0**
**In-hospital diagnostic elucidation**	**23**	**23.5**
**Discovery after death**	**2**	**2.0**
**Urgency**	**16**	**16.3**
**Contacts investigation**	**1**	**1.0**
**Ignored**	**6**	**6.1**
**Outcome**		
**Abandonment**	**4**	**4.1**
**Cure**	**62**	**63.3**
**Bankruptcy/resistance**	**1**	**1.0**
**Death with TB as a basic cause**	**6**	**6.1**
**Death from other causes**	**14**	**14.3**
**Diagnostic change**	**7**	**7.1**
**State/country transfer**	**1**	**1.0**
**Ignored**	**3**	**3.1**
**TB-HIV co-infection**		
**No**	**63**	**64.3**
**Yes**	**24**	**24.5**
**Ignored**	**11**	**11.2**
**Alcoholism**		
**No**	**79**	**80.6**
**Yes**	**19**	**19.4**
**Mental disease**		
**No**	**95**	**96.9**
**Yes**	**3**	**3.1**
**Drug addiction**		
**No**	**94**	**95.9**
**Yes**	**4**	**4.1**
**Smoking**		
**No**	**96**	**98.0**
**Yes**	**2**	**2.0**

*Abbreviations*: *HIV* Human immunodeficiency virus, *TB* Tuberculosis

Regarding the disease profile, most cases were new (*n* = 89, 22.4%), pulmonary TB (*n* = 74, 75.5%) discovered by outpatient demand (*n* = 48, 49.0%), and cure was the outcome (*n* = 62, 63.3%). Regarding the classification of the TB type, concomitant TB means that the disease has an effect on the lungs and other organs (pulmonary or extrapulmonary TB). Of the cases of extrapulmonary TB mentioned in the notification form, there were 4 cases (4.1%) of ganglionic TB, 4 cases (4.1%) of meningeal TB, 1 case (1.0%) of miliary TB, 3 cases (3.1%) of pleural TB, 1 case (1.0%) of TB in the urinary tract, and 3 cases (3.1%) of disseminated TB, which affected multiple organs.

In addition, most cases did not have TB-human immunodeficiency virus (HIV) co-infection (*n* = 63, 64.3%), TB-diabetes co-infection (*n* = 94, 95.9%), alcoholism (*n* = 79; 80.6%), mental illness (*n* = 95, 96.9%), drug addiction (n = 94, 95.9%), or smoking (*n* = 96. 98.0%). It is important to highlight the high number of HIV-positive cases (*n* = 24. 24.5%) and children who reported using alcohol (*n* = 19, 19.4%).

**Fig. 2:**
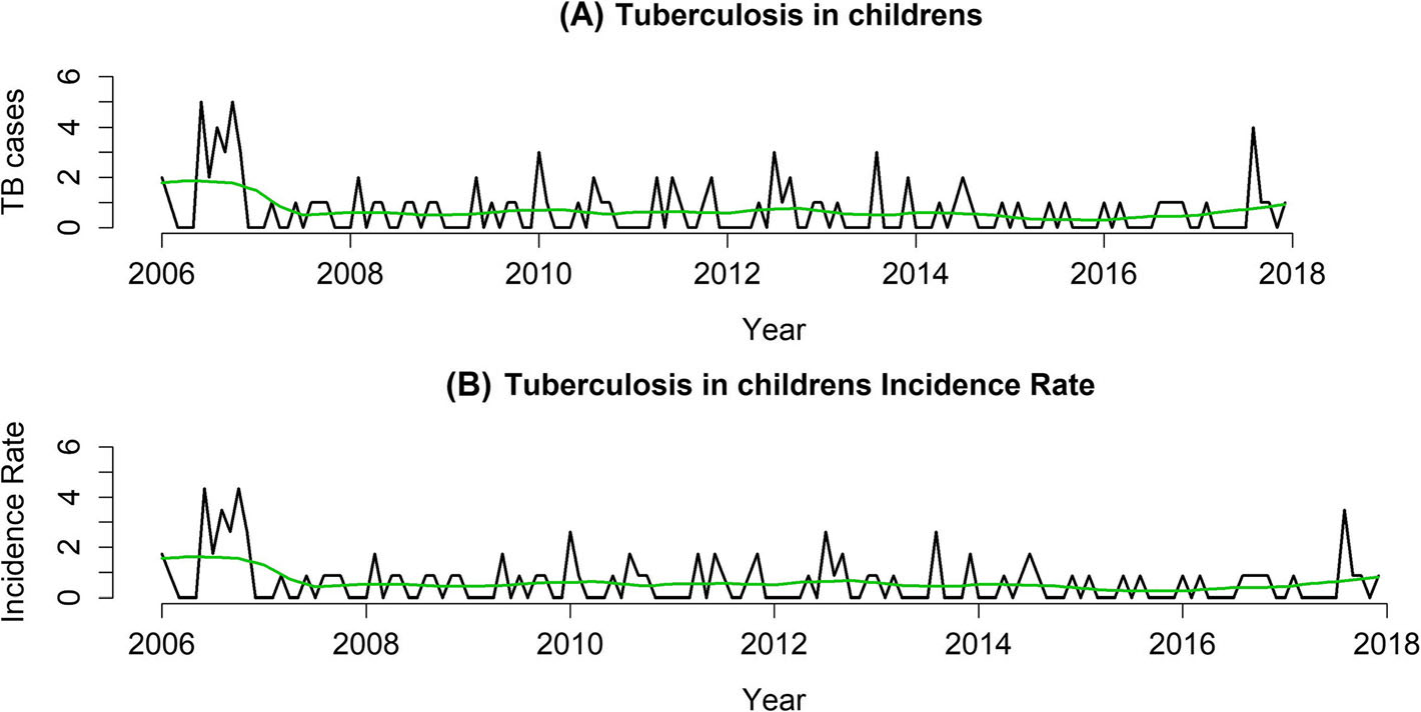
Series and time trend of cases and incidence rates of tuberculosis in children in Ribeirão Preto, São Paulo, Brazil (2006–2017)

Figure 2 shows the behavior of the time series of cases and the incidence rates of TB in children over the study period. There was an apparent stability in which there was little fluctuation in the temporal trend of the observed event with regard to the number cases (Fig. 2a) and incidence rates (Fig. 2b).

Of the 98 total cases of TB in children, 8 cases (8.9%) were excluded due to the lack of address information, such as providing the same street block or complete hospital address as their home address, which made geocoding impossible. Thus, the remaining 90 cases were geocoded and proceeded to the stage of identifying risk areas.

**Fig. 3:**
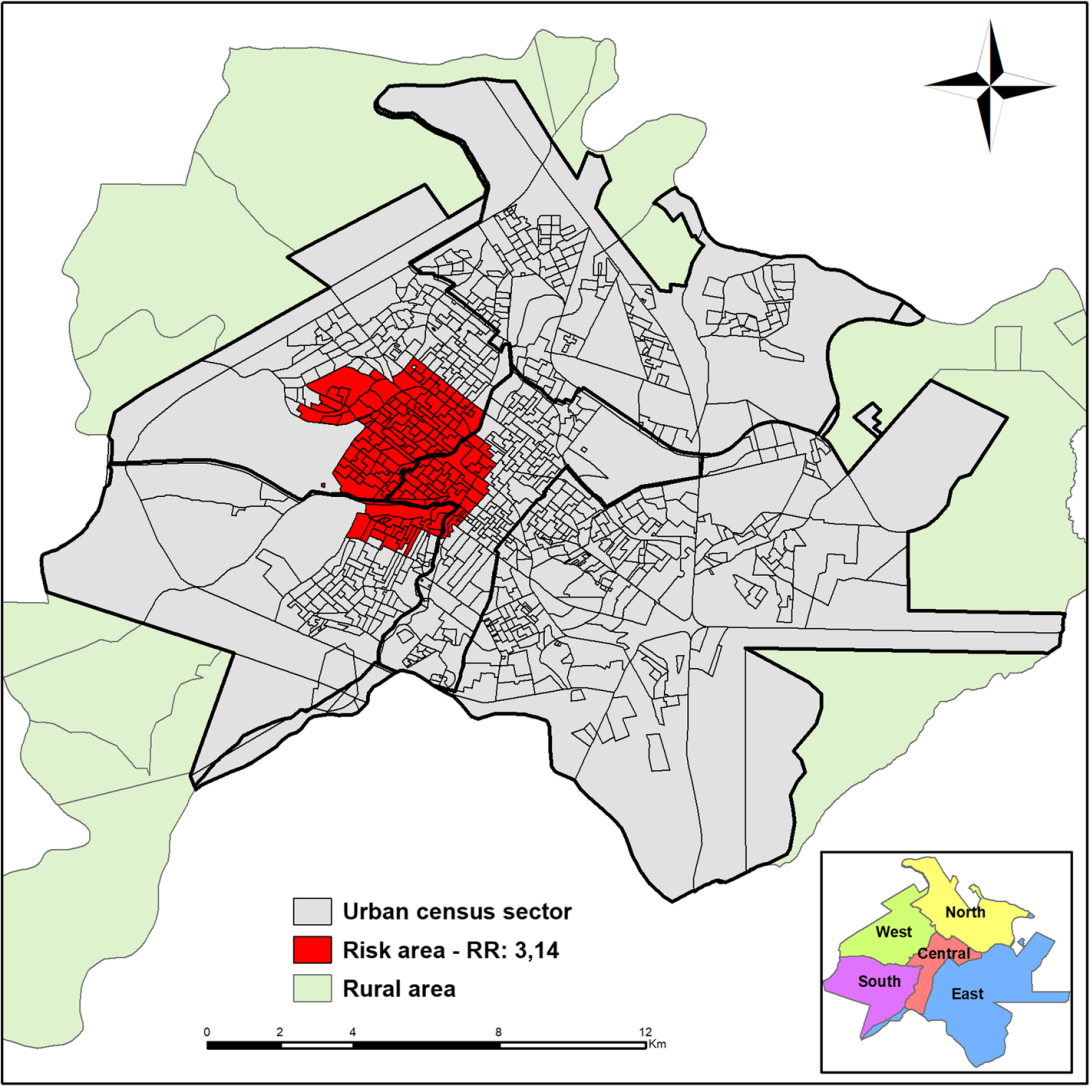
Area of space risk for childhood tuberculosis, Ribeirão Preto (2006–2017)

This analysis identified a space risk area (*p* = 0.006), which shows an OR of 3.14 (95% CI 2.05–4.84) that involves 167 census sections in the central, the western, and the southern districts; 33 cases of childhood TB; and a population of 17,794 children under 15 years of age (Fig. 3).

According to the criteria for the selection of VIFs used for statistical modeling, variables with values greater than 10 were excluded (average number of residents in permanent private housing units, average household income of permanent private housing units, proportion of per capita family income between an eighth and a half minimum wage and proportion of those responsible for individuals 10 to 29 years) (Additional file 1), and 5 variables were excluded due to their total separation, making modeling impossible. Therefore, 14 variables were included in the multiple models. Of the models tested, the ZIP probability distribution was best suited the nature of the variables following the lowest AIC value (581.77).

In the final model (Table 2), there were 8 variables, 6 of them significant: proportion of private versus collective households; proportion of children aged 0 to 5 years in the population; proportion of households living without per capita income; proportion of private households with monthly nominal incomes of up to one quarter of minimum wage levels; proportion of women under 30 years of age responsible as head of household; and measures of average income for women in charge of the household.

**Table 2:** Explanatory model for cases of childhood tuberculosis (Ribeirão Preto, São Paulo, Brazil, 2006–2017) following the zero-inflated Poisson distribution

Variables (median)	Coefficient	P	OR (95% CI)
Private and collective homes (> 85)	1.88	0.005	6.55 (1.75–24.28)
Average residents in permanent private housing units (> 3)	−0.69	0.06	NA
Proportion of children from 0 to 5 years in the population (> 5)	1.13	0.01	3.09 (1.25–7.61)
Proportion of households without per capita income (> 0.6)	1.75	0.01	1.78 (1.12–2.82)
Proportion of private households with monthly nominal incomes of up to 1/4 minimum wages (> 48.6)	1.02	0.02	2.77 (1.10–7.02)
Share of household income in household income (%) (> 69)	−0.53	0.50	NA
Proportion of female heads of household under 30 (> 69)	−1.15	0.04	0.31 (0.10–0.91)
Average income of head of household (> BRL 2344)	−3.19	0.002	0.04 (0.01–0.33)

*Abbreviations*: *BRL* Brazilian real, *CI* Confidence interval, *OR* Odds ratio

In relation to the coefficients of the explanatory variables of the ZIP counting model, the variables identified as risk factors were: number of private and collective households (OR 6.55, 95% CI 1.75–24.28); proportion of children aged 0 to 5 years in the population (OR 3.09, 95% CI, 1.25–7.61); proportion of households below the per capita income (OR 1.78, 95% CI 1.12–2.82); and proportion of private households with nominal monthly incomes only one quarter of minimum wage levels (OR 2.77, 95% CI 1.10–7.02).

Regarding the variables identified as protection factors, there was a proportion of women responsible for the household under 30 years old (OR 0.31, 95% CI 0.10–0.33) and women whose average income was greater than BRL 2344 (OR 0.04, 95% CI 0.01–0.33). Finally, the ZIP distribution model did not identify variables associated with these cases.

## Discussion

This study aimed to identify areas of risk for the diagnosis of TB in children and the possible relationships to the social inequalities noted in areas of Southeast Brazil. It is important to note that children are dependent on an adult for the treatment of TB or any other condition. Thus, it is essential that health professionals engage in a treatment strategy with the responsible caregivers so that there is adherence to the treatment and, consequently, a lower number of abandonments. Furthermore, it is necessary to guide the caregiver about the adverse effects that may occur as a result medications [13]. According to the study conducted with caregivers of children with TB, knowledge about the treatment and pathophysiology of the disease favors treatment adherence [14].

This study also points out that there are several difficulties faced by caregivers, such as guilt from feeling responsible for the child’s illness, fear of death as an outcome, and the suffering and prejudice faced by the lack of knowledge about TB. Caregivers also experience financial difficulties because in most cases one of the parents stops working to accompany the child to treatment. It is crucial for the health team to understand this situation. Caregiver experience contributes to the continuity of treatment and reduces the risk of abandonment prior to completion of interventions [14].

According to this study, the majority of TB cases were new and the most common outcome was cure (63.3%), a rate that is lower than the 85% goal set out by the End TB Strategy, which aims to cure 85% of TB cases by 2035 [15]. The mortality rate due to TB as a basic cause was extremely high (6.1%), a rate that reflects the late diagnosis of the disease. Although, in this study, the majority of TB cases were not related to other clinical factors such as TB-HIV co-infection, mental illness, drug addiction, alcoholism, or smoking, it may be valid to highlight these risk factors.

The use of illicit or licit drugs, such as alcohol, can contribute to a poor prognosis for TB treatment. Dependency on drugs may delay and/or prolong treatment. Drug use may be related to the concomitant process of chemical dependency, which, in addition to the responsibility for taking medications and regular visits to health services, makes it difficult to continue treatment, in addition to forgetting to take the medication and potentiating the hepatotoxic effects [16].

With the use of serial analytical techniques and time trends, the results showed that TB diagnosis in children under 15 years of age had remained stable throughout the study period. However, this trend may indicate that new cases are not being diagnosed and/or reported and, in this context, may serve as an alert for municipal epidemiological surveillance to identify situations in which the reported data may be misrepresentative of true incidences of the disease [17].

More data are required to investigate the health of the adults in close contact with these children to rule out any undiagnosed or untreated TB in the household. Considering that this result represents the reality of the municipality, it still serves as an alert for epidemiological surveillance to investigate the close contacts of these children, as surely there is an undiagnosed or untreated bacilliferous adult.

When analyzing the statistics, there was an area in which children have a 3.14 fold greater risk of TB than in other areas of the municipality. This area is localized in the central, the western, and the southern regions. The central district is one of the oldest districts of the municipality; its most striking feature is the large number of homeless people in the municipality, part of a serious public health problem. The western district, on the other hand, has many slums and many residents per household; the middle economic class predominates. The southern district is the largest group in number of residents with a predominance of the middle-low economic class [18].

The findings lead us to show that children may only be infected through close contact with a person with active bacilliferous TB. Based on the literature, after 14 days of treatment the infected person no longer transmits the disease to others. Therefore, the diagnosis of TB in a child is considered a sentinel event that indicates the presence of people (usually adults) in the child’s environment who are not diagnosed or who are not undergoing treatment for TB.

Our findings follow the results from the study by Cano et al. [3]; those researchers identified an adult with TB in 37.2% of the cases of children diagnosed with TB. Gathering an accurate and current medical and social history of the person with TB allows the identification of household contacts. Contact tracing is considered a key to protect those considered vulnerable to TB and can limit secondary cases (index case contacts) and break the transmission chain of the TB to other patients.

If a person is diagnosed with TB, it becomes necessary for all contacts (including children) to be evaluated by a specialist and, if necessary, initiate treatment for latent TB. This protocol is recommended by the WHO for all infected household contacts, regardless of age [19].

Dodd et al. [20] used mathematical modeling and identified that the management of household contacts, including the detection of secondary cases and latent infection and the carrying out prophylactic treatment, would prevent almost 160,000 cases of TB and almost 110,000 deaths of children under the age of 15 (with most of these preventable deaths for those under 5 years of age living in endemic areas). Therefore, it is essential that the active search for patients be carried out at home and in the places of greatest contact whenever a child is diagnosed with TB.

Of the tested explanatory models, the ZIP model was the one that best fit the data according to the pre-established criterion (lowest AIC value). This model revealed that children living in census sections that have more than 85 private and collective households (OR 6.55), the proportion of households with per capita income lower than 0.6 (OR 1.78), and the proportion of private households with nominal income of up to one quarter of the minimum wage greater than 48.6 (OR 2.77) have a higher risk of contracting TB. The influence of living conditions on the transmission of TB persists and highlights the image of socioeconomic inequalities that result in medical and health inequities [21].

The model also revealed that locations with a proportion of more than 5 children from 0 to 5 years in the population (OR 3.09) is a probable risk factor for TB. This may be related to the immaturity of the immune system itself; studies have shown that environmental and even food antigens induce the body to produce its own immunoglobulins. The child’s immune system is considered immature and more vulnerable to the disease until the maternal antibodies have decreased and his or her own body produces antibodies. This process should occur until the age of 5 years [22]. In 2015, TB caused the death of 210,000 children worldwide, and 80% (191,000) of these deaths occurred in children under the age of 5 years [15]. A systematic review of the subject indicated that changes in TB incidence rates are more associated with variations in socioeconomic indices and consequent change in the general health status of the population than in health services variables [21].

One of the variables identified as protective is the proportion of women responsible for the household under 30 years old and over 69 years old (OR 0.31). This protective factor can be explained, according to research carried out by the Brazilian Institute of Geography and Statistics (IBGE), by the fact that women are having children later [23]. According to a survey conducted between 2008 and 2018 by the IBGE, the number of women under 30 years who had children decreased 16.1%, while the number of women who became mothers after the age of 30 years increased (increase and 36% the number of births for mothers between 30 and 44 years old). In addition, those who had children after the age of 45 years dropped by 14.9% [23]. Thus, it could be deemed reliable to assume that maternal age under 30 years would serve as a protective factor for the child’s resistance to TB.

Also identified as a protective factor for childhood TB contraction is the average income greater than BRL 2344 for women responsible for the household (OR 0.04); this can be justified by the close relationship that TB has with the social determinants of health. A study by San Pedro and Oliveira [21] identified that low income increases vulnerability to TB, reflecting unequal access to information, and unequal access to consumer goods and health services [21]. Conversely, increasing income tends to decrease vulnerability to disease.

Among the limitations of the study are the use of secondary data sources that may lead to incomplete data or writing errors, because the data obtained through the TB notification forms were already tabulated and the researchers had no contact with the participants of this research to verify information. In addition, the main limitation of an ecological study is ecological fallacy: Analyses are carried out at the aggregate level and their results cannot be interpreted at the individual level.

The authors encourage new studies and approaches be carried out to better characterize the relationship between children with TB and cases reported in the same family, as it is a very important and little explored aspect in the literature and new studies should be conducted in order to fill the gaps that still remain on the topic. This study showed areas of risk for the occurrence of TB in children. The study is in line with the End TB Strategy and the 2030 Agenda, which aims to support a strategic agenda for action and, therefore, save the lives of children through the systematic, intensified, and comprehensive identification of children with TB respiratory symptoms in the community.

Therefore, TB continues to be a problem linked to living conditions, as it shows a marked and persistent influence from socioeconomic and cultural factors that worsen the rates of social inequalities and inequities.

## Conclusion

Advances in knowledge by highlighting areas of risk for the occurrence of TB in children and its relationship to the social and economic inequality continues to be crucial to meet the goals of public health initiatives. It is worth mentioning that adults with bacilliferous TB in these regions may still be unknown by health services, so these areas should be treated as priorities for the active search of cases, which may affect epidemiological indicators in the municipality.

## Supplementary information

**Additional file 1.** Table. Variance inflation factor coefficients for cases of childhood tuberculosis and variables of the São Paulo Social Vulnerability Index, Ribeirão Preto, São Paulo, Brazil (2006–2017).

## Data Availability

The data that support the findings of this study are available from Municipal Health Secretariat of Ribeirão Preto but restrictions apply to the availability of these data, which were used under license for the current study, and so are not publicly available. The data were made available to the authors upon request and authorization from Dr. Claudia Siqueira Vassimon, Coordinator of the Research Project Evaluation Commission of the Ribeirão Preto Municipal Health Department.

## Abbreviations

AIC: Akaike information criteria
BN: Binomial negative
BRL: Brazilian real
CI: Confidence interval
HIV: Human immunodeficiency virus
OR: Odds ratio
TB: Tuberculosis
SVG: Social Vulnerability Groups
SVI-SP: Social Vulnerability Index of São Paulo
VIF: Variance inflation factors
WHO: World Health Organization
ZINB: Zero-inflated negative binomial
ZIP: Zero-inflated Poisson

## References

[1] Brasil. Agência Nacional de Vigilância Sanitária. Xpert® MTB/RIF no diagnóstico da tuberculose pulmonar. In: Boletim Brasileiro de Avaliação de Tecnologias em Saúde. 2011. http://bvsms.saude.gov.br/bvs/periodicos/brats_16.pdf. Accessed 30 Sept 2019.

[2] World Health Organization: Global Tuberculosis Report 2019. https://www.who.int/tb/publications/global_report/en/. Accessed 02 Sept 2020.

[3] Cano APG, Romaneli MTN, Pereira RM, Tresoldi AT (2017). Tuberculose em pacientes pediátricos: como tem sido feito o diagnóstico?. Rev Paul Ped.

[4] Ministério da Saúde. Secretaria de Vigilância em Saúde. Departamento de Vigilância das Doenças Transmissíveis. Manual de Recomendações para o Controle da Tuberculose no Brasil / Ministério da Saúde, Secretaria de Vigilância em Saúde, Departamento de Vigilância das Doenças Transmissíveis https://bvsms.saude.gov.br/bvs/publicacoes/manual_recomendacoes_controle_tuberculose_brasil_2_ed.pdf. Accessed 15 Aug 2020.

[5] World Health Organization. Commission on Social Determinants of Health. A conceptual framework for action on the social determinants of health. http://www.who.int/social_determinants/resources/csdh_framework_action_05_07.pdf?ua=1. Accessed 02 Sept 2020.

[6] Acosta LM, Bassanesi SL (2014). The Porto Alegre paradox: social determinants and tuberculosis incidence. Rev Bras Epidemiol.

[7] Instituto Brasileiro de Geografia e Estatística. Censo demográfico 2010: resultados gerais da amostra. http://www.ibge.gov.br/home/estatistica/populacao/censo2010/resultados_gerais_amostra/default_resultados_gerais_amostra.shtm. Accessed 02 Sept 2020.

[8] Cleveland R, Cleveland W, McRee JE (1990). Seasonal-trend decomposition procedure based on LOESS. J Offic Stat.

[9] Kulldorff M, Nagarwalla N (1995). Spatial disease clusters: detection and inference. Stat Med.

[10] Montgomery DC, Peck EA, Vining GG (2012). Introduction to linear regression analysis.

[11] Lambert D (1992). Zero-inflated Poisson regression, with an application to defects in manufacturing. Technometrics..

[12] Wagh YS, Kamalja KK (2018). Zero-inflated models and estimation in zero-inflated Poisson distribution. Commun Stat Simul Comput.

[13] Ministério da Saúde. Secretaria de Vigilância em Saúde. Manual de recomendações para o controle da tuberculose no Brasil http://portal.saude.gov.br/portal/arquivos/pdf/manual_tuberculose.pdf. Accessed 30 Sept 2019.

[14] da Silva TMV, dos Santos MA, Almeida FA (2014). Compreendendo a experiência de cuidadores de crianças com tuberculose em tratamento diretamente observado. Rev Esc Enferm USP.

[15] World Health Organization (2018). National guidelines on management of tuberculosis in children.

[16] Ferreira MRL, Bonfim RO, Sieueira TC, Orfao NH (2018). Abandono do tratamento da tuberculose: uma revisão integrativa. Rev Enferm Contemp.

[17] Rodrigues MAF, Mota ELA. Subnotificação da tuberculose: aplicação da metodologia captura-recaptura. Rev Baiana Saúde Pública. 2017;40. 10.22278/2318-2660.2016.v40.nS2.a2695.

[18] Ribeirão Preto. Prefeitura Municipal. Secretaria Municipal de Saúde. Relação das unidades de saúde https://www.ribeiraopreto.sp.gov.br/ssaude/rede/i16ubs.php. Accessed 30 Sept 2019.

[19] Marais BJ (2018). Preventing tuberculosis in household contacts crucial to protect children and contain epidemic spread. Lancet Glob Health.

[20] Dodd PJ, Yuen CM, Becerra MC, Revill P, Jenkins HE, Seddon JA (2018). Potential effect of household contact management on childhood tuberculosis: a mathematical modelling study. Lancet Glob Health.

[21] San Pedro A, Oliveira RM (2013). Tuberculose e indicadores socioeconômicos: Revisão sistemática da literatura. Rev Panam Salud Pública.

[22] Diniz LMO, Figueiredo BCG (2014). The newborn’s immune system. Revi Médica Minas Gerais.

[23] Instituto Brasileiro de Geografia e Estatística. Sistema de Estatísticas Vitais. https://www.ibge.gov.br/estatisticas/sociais/populacao/9110-estatisticas-do-registro-civil.html?=&t=resultados. Accessed 15 Aug 2020.

