# Supplementary material for: Risk areas for tuberculosis among children and their inequalities in a city from Southeast Brazil
Source: BMC Pediatr. 2020 Oct 6;20:462. doi: 10.1186/s12887-020-02364-7 (PMC7541251; doi:10.1186/s12887-020-02364-7)
Supplement: Supplementary file 1 — Additional file 1. Table. Variance inflation factor coefficients for cases of childhood tuberculosis and variables of the São Paulo Social Vulnerability Index, Ribeirão Preto, São Paulo, Brazil (2006–2017). [file 12887_2020_2364_MOESM1_ESM.docx]

Table - Variance inflation factor coefficients for cases of childhood tuberculosis and variables of the São Paulo Social Vulnerability Index, Ribeirão Preto, São Paulo, Brazil (2006-2017)

| **Variable** | **VIF** |
| --- | --- |
| Private and collective homes | 3.453 |
| Permanent private housing units | 34.229 |
| Total improvised private households | 1.232 |
| Residents in permanent private homes. | 36.713 |
| Average residents in permanent private housing units | 5.386 |
| Proportion of children from 0 to 5 years in the population. | 3.248 |
| Average household income of permanent private housing units | 50.151 |
| Per capita income in permanent private households in the census tract | 24.703 |
| Proportion of households without per capita income | 3.281 |
| Household income ratio per capita up to 1/8 SM | 3.233 |
| Ratio of household income per capita 1/8 to 1/2 SM | 14.349 |
| Proportion of household income per capita from 1/2 to 2 SM | 36.928 |
| Proportion of household income of more than 2 SM | 69.683 |
| Proportion of private households with monthly nominal incomes of up to 1/4 S.M | 7.561 |
| Average age of responsible people. | 12.692 |
| Average household head income | 53.422 |
| Proportion of literate people in charge | 12.933 |
| Proportion of responsible for 10 to 29 years. | 9.552 |
| Share of household income in household income (percentage) | 5.753 |
| Average age of women in charge of the household | 9.667 |
| Proportion of women heads of household literate | 7.688 |
| Proportion of female heads of household under 30 | 7.268 |
| Average income of household heads | 5.286 |

* Dependent variable: cases of childhood TB
